# 2273. Azithromycin Use Before and During COVID- 19 and Impact of Implementing Evidence-based Guidelines

**DOI:** 10.1093/ofid/ofad500.1895

**Published:** 2023-11-27

**Authors:** Sherin Shams, Hanaa Nafady-Hego, Anil G Thomas, Samah Saleem, Fathima Hanana, Aimon Malik, Anvar Hassan Kaleeckal, Ali Nizar Latif, Aftab Mohammad Umar, Abdullatif Alkhal, Muna Almaslamani, Abdul-Badi Abou-Samra, Adeel A Butt

**Affiliations:** Hamad Medical Corporation, Doha, Ad Dawhah, Qatar; Hamad Medical corporation, Doha, Ad Dawhah, Qatar; Hamad Medical corporation, Doha, Ad Dawhah, Qatar; Hamad Medical corporation, Doha, Ad Dawhah, Qatar; Hamad Medical corporation, Doha, Ad Dawhah, Qatar; Hamad Medical corporation, Doha, Ad Dawhah, Qatar; Hamad Medical Corporation, Doha, Ad Dawhah, Qatar; Hamad Medical Corporation, Doha, Ad Dawhah, Qatar; Hamad Medical corporation, Doha, Ad Dawhah, Qatar; Hamad Medical corporation, Doha, Ad Dawhah, Qatar; Communicable Disease Center, Doha, Ad Dawhah, Qatar; Hamad Medical corporation, Doha, Ad Dawhah, Qatar; VA Pittsburgh Healthcare System, Pittsburgh, PA

## Abstract

**Background:**

Early in the COVID-19 pandemic, results from small uncontrolled studies led to azithromycin being proposed as an effective treatment in patients with COVID-19. Numerous subsequent randomized controlled trials and observational studies have failed to demonstrate azithromycin’s effectiveness in COVID-19. The magnitude of azithromycin overuse, particularly for COVID-19 remains undocumented. We sought to quantify the use of azithromycin before and during the COVID-19 pandemic in a national healthcare system where specific evidence-based guidelines were rapidly implemented for judicious use of various suggested COVID-19 treatments.

**Methods:**

The study was conducted at Hamad Medical Corporation in Qatar, which was the single provider of inpatient care for COVID-19 in Qatar. Evidence-based guidelines were implemented in Qatar for use of azithromycin in June 2020. For our study, all azithromycin prescriptions between 2019-2022 were retrieved from the electronic medical records. Number of azithromycin courses prescribed, and rate/100,000 population were calculated and compared over time. A course was defined as any azithromycin prescription with no gap of > 10 days. A course was deemed related to COVID-19 if written within -3 to +10 days of a positive SARS-CoV-2 PCR. A one-way repeated measured analysis of variance was conducted to evaluate the null hypothesis that there was no change in azithromycin use before and during COVID-19.

**Results:**

During the study period, a total of 203,806 azithromycin courses were prescribed for 166,062 individuals. Overall number of courses increased in the first 2 quarters of 2020 (average 12,857/quarter in 2019 to average of 19,297 in Q1-Q2 of 2020) and then dropped precipitously to 9,881/quarter over the next 6 quarters. COVID-19 related azithromycin courses peaked in 2020-Q2 (13,691) and dropped subsequently to 2,836 for 2020-Q3, 1,410 for 2020-Q4; 5,465 for 2021-Q1, and 4,288 for 2021-Q2. Stringent evidence-based guidelines implemented in June 2020 for use of various COVID-19 therapeutics coincided with the rapid drop in azithromycin use for COVID-19.

**Figure:**
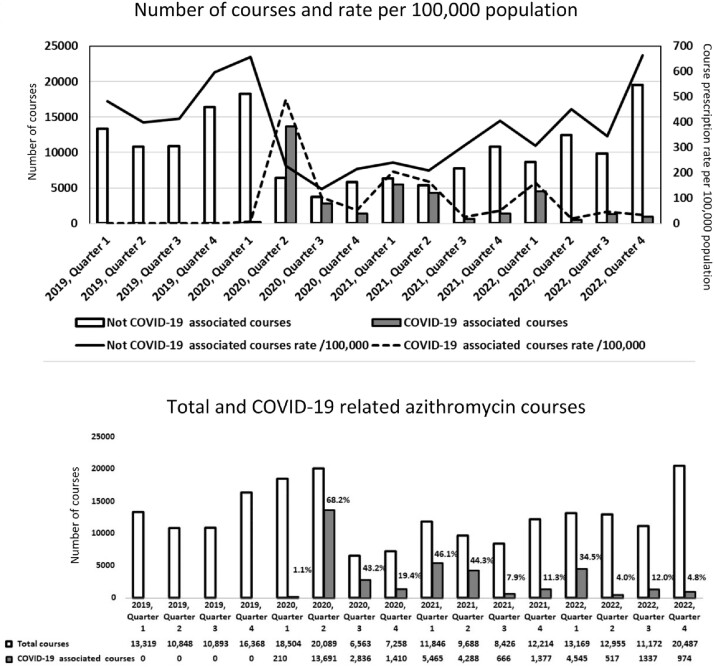

**Conclusion:**

While azithromycin prescriptions increased immediately after the COVID-19 pandemic, a rapid decline was observed immediately after implementing evidence-based guidelines

**Disclosures:**

**Adeel A. Butt, MBBS, MS**, Gilead Sciences: Grant/Research Support|Merck and Company: Grant/Research Support

